# Some generalizations of inequalities for sector matrices

**DOI:** 10.1186/s13660-018-1786-8

**Published:** 2018-07-23

**Authors:** Chaojun Yang, Fangyan Lu

**Affiliations:** Department of Mathematics, Soochow University, Suzhou, P.R. China

**Keywords:** 15A45, 15A15, Sector matrix, Schatten *p*-norm, Accretive-dissipative matrix, Inequality

## Abstract

In this paper, we generalize some Schatten *p*-norm inequalities for accretive-dissipative matrices obtained by Kittaneh and Sakkijha. Moreover, we present some inequalities for sector matrices.

## Introduction

Throughout this paper, let $\mathbb{M}_{n}$ be the set of all $n\times n$ complex matrices. We denote by $I_{n}$ the identity matrix in $\mathbb{M}_{n}$. For two Hermitian matrices $A, B\in\mathbb{M}_{n}$, we use $A\ge B$ ($B\le A$) to mean that $A-B$ is a positive semidefinite matrix. A matrix $A\in\mathbb{M}_{n}$ is called accretive-dissipative if in its Cartesian (or Toeptliz) decomposition, $A=\Re (A)+i \Im (A)$, the matrices $\Re (A)$ and $\Im (A)$ are positive semidefinite, where $\Re(A)=\frac{A+A^{*}}{2}$, $\Im(A)=\frac{A-A^{*}}{2i}$.

Let $|\!|\!|\cdot |\!|\!|$ denote any unitarily invariant norm on $\mathbb{M}_{n}$. Note that tr is the usual trace functional. For $p>0$ and $A\in\mathbb{M}_{n}$, let $\Vert A \Vert _{p}=(\sum_{j=1}^{n} s_{j}^{p}(A))^{\frac{1}{p}}$, where $s_{1}(A)\ge s_{2}(A)\ge\cdots\ge s_{n}(A)$ are the singular values of *A*. Thus, $\Vert A \Vert _{p}=(\operatorname{tr}|A|^{p})^{\frac{1}{p}}$. For $p\ge1$, this is the Schatten *p*-norm of *A*. For more information about the Schatten *p*-norms, see [1, p. 92].

A real-valued continuous function *f* on an interval *I* is called matrix concave of order *n* if $f(\alpha A+(1-\alpha)B)\ge \alpha f(A)+(1-\alpha)f(B)$ for any two Hermitian matrices $A, B\in\mathbb{M}_{n}$ with spectrum in *I* and all $\alpha\in[0,1]$. Furthermore, *f* is called operator concave if *f* is matrix concave for all *n*.

The numerical range of $A\in\mathbb{M}_{n}$ is defined by

$$ W(A)= \bigl\{ x^{*}Ax : x\in\mathbb{C}^{n}, x^{*}x=1 \bigr\} . $$

For $\alpha\in [0,\frac{\pi}{2})$, $S_{\alpha}$ denotes the sector in the complex plane as follows:

$$ S_{\alpha}= \bigl\{ z\in\mathbb{C} : \Re z\ge0, \vert \Im z \vert \le (\Re z)\tan\alpha \bigr\} . $$

Clearly, *A* is positive semidefinite if and only if $W(A)\subset S_{0}$, and if $W(A), W(B)\subset S_{\alpha}$ for some $\alpha\in[0,\frac{\pi}{2})$, then $W(A+B)\subset S_{\alpha}$. As $0\notin S_{\alpha}$, if $W(A)\subset S_{\alpha}$, then *A* is nonsingular.

In [7], Kittaneh and Sakkijha gave the following Schatten-*p* norm inequalities involving sums of accretive-dissipative matrices.

### Theorem 1.1

*Let* $S, T\in\mathbb{M}_{n}$ *be accretive*-*dissipative*. *Then*

$$ 2^{\frac{-p}{2}}\bigl( \Vert S \Vert ^{p}_{p}+ \Vert T \Vert ^{p}_{p}\bigr)\le \Vert S+T \Vert ^{p}_{p}\le2^{\frac{3}{2}p-1}\bigl( \Vert S \Vert ^{p}_{p}+ \Vert T \Vert ^{p}_{p} \bigr) \quad \textit{for }p\ge1 . $$

In [5], Garg and Aujla showed the following inequalities:

1 $$ \prod_{j=1}^{k} s_{j}\bigl( \vert A+B \vert ^{r}\bigr)\le\prod_{j=1}^{k} s_{j}\bigl(I_{n}+ \vert A \vert ^{r}\bigr) \prod_{j=1}^{k} s_{j} \bigl(I_{n}+ \vert B \vert ^{r}\bigr)\quad \text{for }1\le k\le n, 1\le r\le2; $$

and

2 $$ \prod_{j=1}^{k} s_{j} \bigl(I_{n}+f\bigl( \vert A+B \vert \bigr)\bigr)\le\prod _{j=1}^{k} s_{j}\bigl(I_{n}+f \bigl( \vert A \vert \bigr)\bigr)\prod_{j=1}^{k} s_{j}\bigl(I_{n}+f\bigl( \vert B \vert \bigr)\bigr)\quad \text{for }1\le k\le n, $$

where $A, B\in\mathbb{M}_{n}$ and $f : [0,\infty)\rightarrow [0,\infty)$ is an operator concave function.

By letting $A, B\ge0 $, $r=1$ and $f(X)=X$ for any $X\in\mathbb{M}_{n}$ in (1) and (2), we have

3 $$ \prod_{j=1}^{k} s_{j}(A+B)\le \prod_{j=1}^{k} s_{j}(I_{n}+A) \prod_{j=1}^{k} s_{j}(I_{n}+B) \quad \text{for }1\le k\le n; $$

and

4 $$ \prod_{j=1}^{k} s_{j}(I_{n}+A+B) \le\prod_{j=1}^{k} s_{j}(I_{n}+A) \prod_{j=1}^{k} s_{j}(I_{n}+B) \quad \text{for }1\le k\le n. $$

In this paper, we give a generalization of Theorem 1.1. Moreover, we present some inequalities for sector matrices based on (3) and (4) which remove the absolute values in (1) and (2) from the right-hand side.

## Main results

Before we give the main results, let us present the following lemmas that will be useful later.

### Lemma 2.1

([2, 11])

*Let* $A_{1}, \ldots, A_{n}\in\mathbb{M}_{n}$ *be positive semidefinite*. *Then*

$$ \sum_{j=1}^{n} \Vert A_{j} \Vert ^{p}_{p}\le \Biggl\Vert \sum _{j=1}^{n} A_{j} \Biggr\Vert ^{p}_{p}\le n^{p-1}\sum_{j=1}^{n} \Vert A_{j} \Vert ^{p}_{p} \quad \textit{for }p \ge1. $$

### Lemma 2.2

([3])

*Let* $A, B\in\mathbb{M}_{n}$ *be positive semidefinite*. *Then*

$$ \Vert A+iB \Vert _{p}\le \Vert A+B \Vert _{p}\le \sqrt{2} \Vert A+iB \Vert _{p} \quad \textit{for }p\ge1. $$

Our first main result is a generalization of Theorem 1.1.

### Theorem 2.3

*Let* $A_{1}, \ldots, A_{n}\in\mathbb{M}_{n}$ *be accretive*-*dissipative*. *Then*

$$ 2^{\frac{-p}{2}}\sum_{j=1}^{n} \Vert A_{j} \Vert ^{p}_{p}\le \Biggl\Vert \sum _{j=1}^{n} A_{j} \Biggr\Vert ^{p}_{p}\le\frac{(2n^{2})^{\frac{p}{2}}}{n}\sum _{j=1}^{n} \Vert A_{j} \Vert ^{p}_{p} \quad \textit{for }p\ge1. $$

### Proof

Let $A_{j}=B_{j}+iC_{j}$ be the Cartesian decompositions of $A_{j}$, $j=1, \ldots, n$. Then we have

$$\begin{aligned} \Biggl\Vert \sum_{j=1}^{n} A_{j} \Biggr\Vert ^{p}_{p}&= \Biggl\Vert \sum _{j=1}^{n} (B_{j}+iC_{j}) \Biggr\Vert ^{p}_{p} \\ &= \Biggl\Vert \sum_{j=1}^{n} {B_{j}}+i\sum_{j=1}^{n}{C_{j}} \Biggr\Vert ^{p}_{p} \\ &\ge2^{\frac{-p}{2}} \Biggl\Vert \sum_{j=1}^{n} {B_{j}}+\sum_{j=1}^{n}{C_{j}} \Biggr\Vert ^{p}_{p}\quad\text{(by Lemma 2.2)} \\ &=2^{\frac{-p}{2}} \Biggl\Vert \sum_{j=1}^{n} (B_{j}+C_{j}) \Biggr\Vert ^{p}_{p} \\ &\ge2^{\frac{-p}{2}}\sum_{j=1}^{n} \Vert B_{j}+C_{j} \Vert ^{p}_{p} \quad \text{(by Lemma 2.1)} \\ &\ge2^{\frac{-p}{2}}\sum_{j=1}^{n} \Vert B_{j}+iC_{j} \Vert ^{p}_{p} \quad \text{(by Lemma 2.2)} \\ &=2^{\frac{-p}{2}}\sum_{j=1}^{n} \Vert A_{j} \Vert ^{p}_{p}, \end{aligned}$$

which proves the first inequality.

To prove the second inequality, compute

$$\begin{aligned} \Biggl\Vert \sum_{j=1}^{n} A_{j} \Biggr\Vert ^{p}_{p}&= \Biggl\Vert \sum _{j=1}^{n} (B_{j}+iC_{j}) \Biggr\Vert ^{p}_{p} \\ &= \Biggl\Vert \sum_{j=1}^{n} {B_{j}}+i\sum_{j=1}^{n}{C_{j}} \Biggr\Vert ^{p}_{p} \\ &\le \Biggl\Vert \sum_{j=1}^{n} {B_{j}}+\sum_{j=1}^{n}{C_{j}} \Biggr\Vert ^{p}_{p}\quad\text{(by Lemma 2.2)} \\ &= \Biggl\Vert \sum_{j=1}^{n} (B_{j}+C_{j}) \Biggr\Vert ^{p}_{p} \\ &\le n^{p-1}\sum_{j=1}^{n} \Vert B_{j}+C_{j} \Vert ^{p}_{p} \quad \text{(by Lemma 2.1)} \\ &\le n^{p-1}2^{\frac{p}{2}}\sum_{j=1}^{n} \Vert B_{j}+iC_{j} \Vert ^{p}_{p} \quad\text{(by Lemma 2.2)} \\ &=\frac{(2n^{2})^{\frac{p}{2}}}{n}\sum_{j=1}^{n} \Vert A_{j} \Vert ^{p}_{p}, \end{aligned}$$

which completes the proof. □

### Remark 2.4

By letting $n=2$ in Theorem 2.3, we thus get Theorem 1.1.

The following lemma is the well-known Fan–Hoffman inequality.

### Lemma 2.5

([12, p. 63])

*Let* $A\in\mathbb{M}_{n}$. *Then*

$$ \lambda_{j}(\Re A)\le s_{j}(A), $$

*where* $\lambda_{j}(\cdot)$ *denotes the* *jth largest eigenvalue*.

In [4], Drury and Lin presented a reverse version of Lemma 2.5 as follows.

### Lemma 2.6

*Let* $A\in\mathbb{M}_{n}$ *be such that* $W(A)\subset S_{\alpha}$. *Then*

$$ s_{j}(A)\le \sec^{2}(\alpha)\lambda_{j}(\Re A), $$

*where* $\lambda_{j}(\cdot)$ *denotes the* *jth largest eigenvalue*.

### Theorem 2.7

*Let* $A, B\in\mathbb{M}_{n}$ *be such that* $W(A), W(B)\subset S_{\alpha}$. *Then*

5 $$ \prod_{j=1}^{k} s_{j}(A+B)\le \sec^{2k}(\alpha)\prod_{j=1}^{k} s_{j}(I_{n}+A)\prod_{j=1}^{k} s_{j}(I_{n}+B)\quad \textit{for }1\le k\le n; $$

*and*

6 $$ \prod_{j=1}^{k} s_{j}(I_{n}+A+B) \le\sec^{2k}(\alpha)\prod_{j=1}^{k} s_{j}(I_{n}+A)\prod_{j=1}^{k} s_{j}(I_{n}+B)\quad \textit{for }1\le k\le n. $$

### Proof

We have

$$\begin{aligned} \prod_{j=1}^{k} s_{j}(A+B)&\le \sec^{2k}(\alpha)\prod_{j=1}^{k} s_{j}\bigl(\Re(A+B)\bigr)\quad\text{(by Lemma 2.6)} \\ &=\sec^{2k}(\alpha)\prod_{j=1}^{k} s_{j}\bigl(\Re(A)+\Re(B)\bigr) \\ &\le\sec^{2k}(\alpha)\prod_{j=1}^{k} s_{j}\bigl(I_{n}+\Re(A)\bigr)\prod _{j=1}^{k} s_{j}\bigl(I_{n}+ \Re(B)\bigr)\quad\bigl(\text{by (3)}\bigr) \\ &=\sec^{2k}(\alpha)\prod_{j=1}^{k} s_{j}\bigl(\Re(I_{n}+A)\bigr)\prod _{j=1}^{k} s_{j}\bigl(\Re(I_{n}+B) \bigr) \\ &\le\sec^{2k}(\alpha)\prod_{j=1}^{k} s_{j}(I_{n}+A)\prod_{j=1}^{k} s_{j}(I_{n}+B)\quad\text{(by Lemma 2.5)} \end{aligned}$$

which proves (5).

To prove (6), compute

$$\begin{aligned} \prod_{j=1}^{k} s_{j}(I_{n}+A+B)& \le\sec^{2k}(\alpha)\prod_{j=1}^{k} s_{j}\bigl(\Re(I_{n}+A+B)\bigr)\quad\text{(by Lemma 2.6)} \\ &=\sec^{2k}(\alpha)\prod_{j=1}^{k} s_{j}\bigl(I_{n}+\Re(A)+\Re(B)\bigr) \\ &\le\sec^{2k}(\alpha)\prod_{j=1}^{k} s_{j}\bigl(I_{n}+\Re(A)\bigr)\prod _{j=1}^{k} s_{j}\bigl(I_{n}+ \Re(B)\bigr)\quad\bigl(\text{by (4)}\bigr) \\ &=\sec^{2k}(\alpha)\prod_{j=1}^{k} s_{j}\bigl(\Re(I_{n}+A)\bigr)\prod _{j=1}^{k} s_{j}\bigl(\Re(I_{n}+B) \bigr) \\ &\le\sec^{2k}(\alpha)\prod_{j=1}^{k} s_{j}(I_{n}+A)\prod_{j=1}^{k} s_{j}(I_{n}+B),\quad\text{(by Lemma 2.5)} \end{aligned}$$

which completes the proof. □

### Corollary 2.8

*Let* $A, B\in\mathbb{M}_{n}$ *be such that* $W(A), W(B)\subset S_{\alpha}$. *Then*, *for all unitarily invariant norms* $|\!|\!|\cdot |\!|\!|$ *on* $\mathbb{M}_{n}$,

$$ |\!|\!|A+B |\!|\!|\le\sec^{2}(\alpha) |\!|\!|I_{n}+A |\!|\!||\!|\!|I_{n}+B |\!|\!|; $$

*and*

$$ |\!|\!|I_{n}+A+B |\!|\!|\le\sec^{2}(\alpha) |\!|\!|I_{n}+A |\!|\!||\!|\!|I_{n}+B |\!|\!|. $$

### Proof

From (5) and (6), we obtain

$$ \prod_{j=1}^{k} s_{j}(A+B)\le \prod_{j=1}^{k} s_{j}\bigl(\sec( \alpha) (I_{n}+A)\bigr)s_{j}\bigl(\sec(\alpha) (I_{n}+B)\bigr)\quad \text{for }1\le k\le n; $$

and

$$ \prod_{j=1}^{k} s_{j}(I_{n}+A+B) \le\prod_{j=1}^{k} s_{j}\bigl( \sec(\alpha) (I_{n}+A)\bigr)s_{j}\bigl(\sec(\alpha) (I_{n}+B)\bigr)\quad \text{for }1\le k\le n, $$

which is equivalent to the following inequalities:

$$ \prod_{j=1}^{k} s_{j}^{\frac{1}{2}}(A+B) \le\prod_{j=1}^{k} s_{j}^{\frac{1}{2}} \bigl(\sec(\alpha) (I_{n}+A)\bigr)s_{j}^{\frac{1}{2}}\bigl( \sec(\alpha) (I_{n}+B)\bigr)\quad \text{for }1\le k\le n; $$

and

$$ \prod_{j=1}^{k} s_{j}^{\frac{1}{2}}(I_{n}+A+B) \le\prod_{j=1}^{k} s_{j}^{\frac{1}{2}} \bigl(\sec(\alpha) (I_{n}+A)\bigr)s_{j}^{\frac{1}{2}}\bigl( \sec(\alpha) (I_{n}+B)\bigr)\quad \text{for }1\le k\le n. $$

By the property of majorization [1, p. 42], we have

$$ \sum_{j=1}^{k} s_{j}^{\frac{1}{2}}(A+B) \le\sum_{j=1}^{k} s_{j}^{\frac{1}{2}} \bigl(\sec(\alpha) (I_{n}+A)\bigr)s_{j}^{\frac{1}{2}}\bigl( \sec(\alpha) (I_{n}+B)\bigr)\quad \text{for }1\le k\le n; $$

and

$$ \sum_{j=1}^{k} s_{j}^{\frac{1}{2}}(I_{n}+A+B) \le\sum_{j=1}^{k} s_{j}^{\frac{1}{2}} \bigl(\sec(\alpha) (I_{n}+A)\bigr)s_{j}^{\frac{1}{2}}\bigl( \sec(\alpha) (I_{n}+B)\bigr)\quad \text{for }1\le k\le n. $$

Now, by the Cauchy–Schwarz inequality, we obtain

$$ \sum_{j=1}^{k} s_{j}^{\frac{1}{2}}(A+B) \le\Biggl(\sum_{j=1}^{k} s_{j} \bigl(\sec(\alpha) (I_{n}+A)\bigr)\Biggr)^{\frac{1}{2}}\Biggl(\sum _{j=1}^{k}s_{j}\bigl(\sec(\alpha) (I_{n}+B)\bigr)\Biggr)^{\frac{1}{2}}\quad \text{for }1\le k\le n; $$

and

$$ \begin{aligned} &\sum_{j=1}^{k} s_{j}^{\frac{1}{2}}(I_{n}+A+B) \le\Biggl(\sum_{j=1}^{k} s_{j} \bigl(\sec(\alpha) (I_{n}+A)\bigr)\Biggr)^{\frac{1}{2}}\Biggl(\sum _{j=1}^{k}s_{j}\bigl(\sec(\alpha) (I_{n}+B)\bigr)\Biggr)^{\frac{1}{2}}\\ &\quad \text{for }1\le k\le n, \end{aligned} $$

which is equivalent to the following inequalities:

$$ \bigl\Vert \vert A+B \vert ^{\frac{1}{2}} \bigr\Vert ^{2}_{k}\le \bigl\Vert \sec(\alpha) (I_{n}+A) \bigr\Vert _{k} \bigl\Vert \sec(\alpha) (I_{n}+B) \bigr\Vert _{k}; $$

and

$$ \bigl\Vert \vert I_{n}+A+B \vert ^{\frac{1}{2}} \bigr\Vert ^{2}_{k}\le \bigl\Vert \sec(\alpha) (I_{n}+A) \bigr\Vert _{k} \bigl\Vert \sec(\alpha) (I_{n}+B) \bigr\Vert _{k}. $$

By the generalization of Fan dominance theorem [8], we have

7 $$ \bigl|\!\bigl|\!\bigl|\vert A+B \vert ^{\frac{1}{2}} \bigr|\!\bigr|\!\bigr|^{2} \le \bigl|\!\bigl|\!\bigl|\sec(\alpha) (I_{n}+A) \bigr|\!\bigr|\!\bigr|\bigl|\!\bigl|\!\bigl|\sec(\alpha) (I_{n}+B) \bigr|\!\bigr|\!\bigr|; $$

and

8 $$ \bigl|\!\bigl|\!\bigl|\vert I_{n}+A+B \vert ^{\frac{1}{2}} \bigr|\!\bigr|\!\bigr|^{2}\le \bigl|\!\bigl|\!\bigl|\sec(\alpha) (I_{n}+A) \bigr|\!\bigr|\!\bigr|\bigl|\!\bigl|\!\bigl|\sec(\alpha) (I_{n}+B) \bigr|\!\bigr|\!\bigr|. $$

Let $A+B=U|A+B|$, $I_{n}+A+B=V|I_{n}+A+B|$ be the polar decomposition of $A+B$ and $I_{n}+A+B$, respectively, where *U* and *V* are unitary matrices. Thus, by (7), we have

$$\begin{aligned} |\!|\!|A+B |\!|\!|&= \bigl|\!\bigl|\!\bigl|U \vert A+B \vert \bigr|\!\bigr|\!\bigr|\\ &= \bigl|\!\bigl|\!\bigl|\bigl( \vert A+B \vert ^{\frac{1}{2}}\bigr)^{2} \bigr|\!\bigr|\!\bigr|\\ &\le \bigl|\!\bigl|\!\bigl|\vert A+B \vert ^{\frac{1}{2}} \bigr|\!\bigr|\!\bigr|^{2} \\ &\le \bigl|\!\bigl|\!\bigl|\sec(\alpha) (I_{n}+A) \bigr|\!\bigr|\!\bigr|\bigl|\!\bigl|\!\bigl|\sec(\alpha) (I_{n}+B) \bigr|\!\bigr|\!\bigr|\\ &=\sec(\alpha)^{2} \bigl|\!\bigl|\!\bigl|(I_{n}+A) \bigr|\!\bigr|\!\bigr|\bigl|\!\bigl|\!\bigl|(I_{n}+B) \bigr|\!\bigr|\!\bigr|. \end{aligned}$$

Similarly, by (8) we have

$$ |\!|\!|I_{n}+A+B |\!|\!|\le\sec^{2}(\alpha) |\!|\!|I_{n}+A |\!|\!||\!|\!|I_{n}+B |\!|\!|, $$

which completes the proof. □

Taking $k=n$ in Theorem 2.7, we obtain the following corollary.

### Corollary 2.9

*Let* $A, B\in\mathbb{M}_{n}$ *be such that* $W(A), W(B)\subset S_{\alpha}$. *Then*

$$ \bigl\vert \det(A+B) \bigr\vert \le\sec^{2n}(\alpha) \bigl\vert \det(I_{n}+A) \bigr\vert \bigl\vert \det(I_{n}+B) \bigr\vert ; $$

*and*

$$ \bigl\vert \det(I_{n}+A+B) \bigr\vert \le\sec^{2n}( \alpha) \bigl\vert \det(I_{n}+A) \bigr\vert \bigl\vert \det(I_{n}+B) \bigr\vert . $$

### Lemma 2.10

([13])

*Let* $A\in\mathbb{M}_{n}$ *be such that* $W(A)\subset S_{\alpha}$. *Then*, *for all unitarily invariant norms* $|\!|\!|\cdot |\!|\!|$ *on* $\mathbb{M}_{n}$,

$$ |\!|\!|A |\!|\!|\le \sec(\alpha) \bigl|\!\bigl|\!\bigl|\Re(A) \bigr|\!\bigr|\!\bigr|. $$

Next we give an improvement of Corollary 2.8.

### Theorem 2.11

*Let* $A, B\in\mathbb{M}_{n}$ *be such that* $W(A), W(B)\subset S_{\alpha}$. *Then*, *for all unitarily invariant norms* $|\!|\!|\cdot |\!|\!|$ *on* $\mathbb{M}_{n}$,

9 $$ |\!|\!|A+B |\!|\!|\le\sec(\alpha) |\!|\!|I_{n}+A |\!|\!||\!|\!|I_{n}+B |\!|\!|; $$

*and*

10 $$ |\!|\!|I_{n}+A+B |\!|\!|\le\sec(\alpha) |\!|\!|I_{n}+A |\!|\!||\!|\!|I_{n}+B |\!|\!|. $$

### Proof

By (3), (4), and the proof of Corollary 2.8, we obtain

11 $$ \bigl|\!\bigl|\!\bigl|\Re(A)+\Re(B) \bigr|\!\bigr|\!\bigr|\le \bigl|\!\bigl|\!\bigl|I_{n}+ \Re(A) \bigr|\!\bigr|\!\bigr|\bigl|\!\bigl|\!\bigl|I_{n}+\Re(B) \bigr|\!\bigr|\!\bigr|; $$

and

12 $$ \bigl|\!\bigl|\!\bigl|I_{n}+\Re(A)+\Re(B) \bigr|\!\bigr|\!\bigr|\le \bigl|\!\bigl|\!\bigl|I_{n}+\Re(A) \bigr|\!\bigr|\!\bigr|\bigl|\!\bigl|\!\bigl|I_{n}+\Re(B) \bigr|\!\bigr|\!\bigr|. $$

Hence

$$\begin{aligned} |\!|\!|A+B |\!|\!|&\le\sec(\alpha) \bigl|\!\bigl|\!\bigl|\Re(A+B) \bigr|\!\bigr|\!\bigr|\quad\text{(by Lemma 2.10)} \\ &=\sec(\alpha) \bigl|\!\bigl|\!\bigl|\Re(A)+\Re(B) \bigr|\!\bigr|\!\bigr|\\ &\le\sec(\alpha) \bigl|\!\bigl|\!\bigl|I_{n}+\Re(A) \bigr|\!\bigr|\!\bigr|\bigl|\!\bigl|\!\bigl|I_{n}+\Re(B) \bigr|\!\bigr|\!\bigr|\quad\bigl(\text{by (11) }\bigr) \\ &=\sec(\alpha) \bigl|\!\bigl|\!\bigl|\Re(I_{n}+A) \bigr|\!\bigr|\!\bigr|\bigl|\!\bigl|\!\bigl|\Re(I_{n}+B) \bigr|\!\bigr|\!\bigr|\\ &\le\sec(\alpha) |\!|\!|I_{n}+A |\!|\!||\!|\!|I_{n}+B |\!|\!|, \end{aligned}$$

which proves (9).

To prove (10), compute

$$\begin{aligned} |\!|\!|I_{n}+A+B |\!|\!|&\le\sec(\alpha) \bigl|\!\bigl|\!\bigl|\Re(I_{n}+A+B) \bigr|\!\bigr|\!\bigr|\quad\text{(by Lemma 2.10)} \\ &=\sec(\alpha) \bigl|\!\bigl|\!\bigl|I_{n}+\Re(A)+\Re(B) \bigr|\!\bigr|\!\bigr|\\ &\le\sec(\alpha) \bigl|\!\bigl|\!\bigl|I_{n}+\Re(A) \bigr|\!\bigr|\!\bigr|\bigl|\!\bigl|\!\bigl|I_{n}+\Re(B) \bigr|\!\bigr|\!\bigr|\quad\bigl(\text{by (12) }\bigr) \\ &=\sec(\alpha) \bigl|\!\bigl|\!\bigl|\Re(I_{n}+A) \bigr|\!\bigr|\!\bigr|\bigl|\!\bigl|\!\bigl|\Re(I_{n}+B) \bigr|\!\bigr|\!\bigr|\\ &\le\sec(\alpha) |\!|\!|I_{n}+A |\!|\!||\!|\!|I_{n}+B |\!|\!|, \end{aligned}$$

which completes the proof. □

The following lemma can be obtained by Lemma 2.5.

### Lemma 2.12

([6, p. 510])

*If* $A\in\mathbb{M}_{n}$ *has positive definite real part*, *then*

$$ \det{(\Re A)}\le \vert \det{A} \vert . $$

### Lemma 2.13

([10])

*Let* $A\in\mathbb{M}_{n}$ *be such that* $W(A)\subset S_{\alpha}$. *Then*

$$ \sec^{n}(\alpha)\det(\Re A)\ge \vert \det A \vert . $$

Now we are ready to give an improvement of Corollary 2.9.

### Theorem 2.14

*Let* $A, B\in\mathbb{M}_{n}$ *be such that* $W(A), W(B)\subset S_{\alpha}$. *Then*

13 $$ \bigl\vert \det(A+B) \bigr\vert \le\sec^{n}(\alpha) \bigl\vert \det(I_{n}+A) \bigr\vert \bigl\vert \det(I_{n}+B) \bigr\vert ; $$

*and*

14 $$ \bigl\vert \det(I_{n}+A+B) \bigr\vert \le\sec^{n}( \alpha) \bigl\vert \det(I_{n}+A) \bigr\vert \bigl\vert \det(I_{n}+B) \bigr\vert . $$

### Proof

Letting $k=n$ in (3) and (4), we have

15 $$ \det\bigl(\Re(A)+\Re(B)\bigr)\le\det\bigl(I_{n}+\Re(A)\bigr) \det \bigl(I_{n}+\Re(B)\bigr); $$

and

16 $$ \det\bigl(I_{n}+\Re(A)+\Re(B)\bigr)\le\det\bigl(I_{n}+\Re(A) \bigr) \det\bigl(I_{n}+\Re(B)\bigr). $$

Thus

$$\begin{aligned} \bigl\vert \det(A+B) \bigr\vert &\le\sec^{n}(\alpha)\det\bigl( \Re(A+B)\bigr)\quad\text{(by Lemma 2.13)} \\ &=\sec^{n}(\alpha)\det\bigl(\Re(A)+\Re(B)\bigr) \\ &\le\sec^{n}(\alpha)\det\bigl(I_{n}+\Re(A)\bigr) \det \bigl(I_{n}+\Re(B)\bigr)\quad\bigl(\text{by (15)}\bigr) \\ &=\sec^{n}(\alpha)\det\bigl(\Re(I_{n}+A)\bigr) \det\bigl( \Re(I_{n}+B)\bigr) \\ &\le\sec^{n}(\alpha) \bigl\vert \det(I_{n}+A) \bigr\vert \bigl\vert \det(I_{n}+B) \bigr\vert \quad\text{(by Lemma 2.12)} \end{aligned}$$

which proves (13).

To prove (14), compute

$$\begin{aligned} \bigl\vert \det(I_{n}+A+B) \bigr\vert &\le\sec^{n}( \alpha)\det\bigl(\Re(I_{n}+A+B)\bigr)\quad\text{(by Lemma 2.13)} \\ &=\sec^{n}(\alpha)\det\bigl(I_{n}+\Re(A)+\Re(B)\bigr) \\ &\le\sec^{n}(\alpha)\det\bigl(I_{n}+\Re(A)\bigr) \det \bigl(I_{n}+\Re(B)\bigr)\quad\bigl(\text{by (16)}\bigr) \\ &=\sec^{n}(\alpha)\det\bigl(\Re(I_{n}+A)\bigr) \det\bigl( \Re(I_{n}+B)\bigr) \\ &\le\sec^{n}(\alpha) \bigl\vert \det(I_{n}+A) \bigr\vert \bigl\vert \det(I_{n}+B) \bigr\vert \quad\text{(by Lemma 2.12)} \end{aligned}$$

which completes the proof. □

### Lemma 2.15

([9])

*Let* $A, B\in\mathbb{M}_{n}$ *be positive semidefinite*. *Then*

$$ \bigl\vert \det{(A+iB)} \bigr\vert \le\det{(A+B)}\le2^{\frac{n}{2}} \bigl\vert \det{(A+iB)} \bigr\vert . $$

We remark that (2) extends the well-known Rotfel’d inequality:

17 $$ \det{\bigl(I_{n}+\mu \vert A+B \vert ^{p}\bigr)}\le\det{ \bigl(I_{n}+\mu \vert A \vert ^{p}\bigr)} \det{ \bigl(I_{n}+\mu \vert B \vert ^{p}\bigr)}\quad\text{for } \mu>0, 0\le p\le1. $$

Finally, we present two inequalities for accretive-dissipative matrices.

### Theorem 2.16

*Let* $A, B\in\mathbb{M}_{n}$ *be accretive*-*dissipative and* $\mu>0$. *Then*

18 $$ \bigl\vert \det{(A+B)} \bigr\vert \le2^{n} \bigl\vert \det{(I_{n}+A)} \bigr\vert \bigl\vert \det{(I_{n}+B)} \bigr\vert ; $$

*and*

19 $$ \bigl\vert \det{\bigl(I_{n}+\mu(A+B)\bigr)} \bigr\vert \le2^{n} \bigl\vert \det{(I_{n}+\mu A)} \bigr\vert \bigl\vert \det{(I_{n}+\mu B)} \bigr\vert . $$

*In particular*,

$$ \bigl\vert \det{(I_{n}+A+B)} \bigr\vert \le2^{n} \bigl\vert \det{(I_{n}+A)} \bigr\vert \bigl\vert \det{(I_{n}+B)} \bigr\vert . $$

### Proof

Let $A=A_{1}+iA_{2}$ and $B=B_{1}+iB_{2}$ be the Cartesian decompositions of *A* and *B*. By (3) and (17), we obtain

20 $$ \det{(A_{1}+A_{2}+B_{1}+B_{2})}\le \det{(I_{n}+A_{1}+A_{2})} \det{(I_{n}+B_{1}+B_{2})}; $$

and

21 $$ \det\bigl({I_{n}+\mu(A_{1}+A_{2}+B_{1}+B_{2})} \bigr)\le\det{\bigl(I_{n}+\mu(A_{1}+A_{2})\bigr)} \det{\bigl(I_{n}+\mu(B_{1}+B_{2})\bigr)}. $$

Hence

$$\begin{aligned} \bigl\vert \det{(A+B)} \bigr\vert &= \bigl\vert \det{(A_{1}+iA_{2}+B_{1}+iB_{2})} \bigr\vert \\ &= \bigl\vert \det{\bigl((A_{1}+B_{1})+i(A_{2}+B_{2}) \bigr)} \bigr\vert \\ &\le\det{(A_{1}+B_{1}+A_{2}+B_{2})} \quad\text{(by Lemma 2.15)} \\ &=\det{(A_{1}+A_{2}+B_{1}+B_{2})} \\ &\le\det{(I_{n}+A_{1}+A_{2})} \det{(I_{n}+B_{1}+B_{2})}\quad\bigl(\text{by (20)}\bigr) \\ &\le2^{n} \bigl\vert \det{(I_{n}+A_{1}+iA_{2})} \bigr\vert \bigl\vert \det{(I_{n}+B_{1}+iB_{2})} \bigr\vert \quad\text{(by Lemma 2.15)} \\ &=2^{n} \bigl\vert \det{(I_{n}+A)} \bigr\vert \bigl\vert \det{(I_{n}+B)} \bigr\vert , \end{aligned}$$

which proves (18).

To prove (19), compute

$$\begin{aligned} &\bigl\vert \det{\bigl(I_{n}+\mu(A+B)\bigr)} \bigr\vert \\ &\quad = \bigl\vert \det{\bigl(I_{n}+\mu(A_{1}+iA_{2}+B_{1}+iB_{2}) \bigr)} \bigr\vert \\ &\quad = \bigl\vert \det{\bigl(I_{n}+\mu(A_{1}+B_{1})+ \mu i(A_{2}+B_{2})\bigr)} \bigr\vert \\ &\quad \le\det{\bigl(I_{n}+\mu(A_{1}+B_{1}+A_{2}+B_{2}) \bigr)}\quad\text{(by Lemma 2.15)} \\ &\quad =\det{\bigl(I_{n}+\mu(A_{1}+A_{2}+B_{1}+B_{2}) \bigr)} \\ &\quad \le\det{\bigl(I_{n}+\mu(A_{1}+A_{2})\bigr)} \det{\bigl(I_{n}+\mu(B_{1}+B_{2})\bigr)}\quad\bigl(\text{ by (21)}\bigr) \\ &\quad \le2^{n} \bigl\vert \det{\bigl(I_{n}+ \mu(A_{1}+iA_{2})\bigr)} \bigr\vert \bigl\vert \det{ \bigl(I_{n}+\mu(B_{1}+iB_{2})\bigr)} \bigr\vert \quad\text{(by Lemma 2.15)} \\ &\quad =2^{n} \bigl\vert \det{(I_{n}+\mu A)} \bigr\vert \bigl\vert \det{(I_{n}+\mu B)} \bigr\vert , \end{aligned}$$

which completes the proof. □
